# Recruitment using respondent driven sampling, risk behaviors assessment and willingness of young female sex workers (18–25 years) in Dar Es Salaam, Tanzania to participate in HIV vaccine trials

**DOI:** 10.1186/s12889-019-7822-x

**Published:** 2019-11-20

**Authors:** Theodora Mbunda, Edith A. M. Tarimo, Muhammad Bakari, Eric Sandström, Asli Kulane

**Affiliations:** 1 Department of Internal Medicine, School of Medicine, Muhimbili University of Health and Allied Sciences, Dar es Salaam, Tanzania; 2 Equity and Health Policy Research Group, Department of Public Health Sciences, Karolinska Institutet, Stockholm, Sweden; 3 Department of Nursing Management, School of Nursing, Muhimbili University of Health and Allied Sciences, Dar es Salaam, Tanzania; 4 Management and Development for Health, Dar es Salaam, Tanzania; 5 Department of Clinical Science and Education, Södersjukhuset, Karolinska Institutet, Stockholm, Sweden

## Abstract

**Background:**

Despite the present HIV preventive and treatment programs, the prevalence of HIV is still high in eastern and southern Africa, among young women and populations at high.

risk for HIV transmission such as sex workers. There is a need to prepare a suitable population that will participate in efficacy HIV vaccine trials to determine the efficacy of HIV vaccines that had proven to be safe and immune potent.

**Methods:**

It was a cross-sectional study that recruited 600 female sex workers using respondent-driven sampling in Dar es Salaam. The study examined recruitment approaches, risk behaviors and willingness of young female sex workers to participate in an HIV vaccine trial. Descriptive statistics described risk behaviors and willingness of the participants to participate in efficacy HIV vaccine trials. The logistic regression model computed the likelihood of willingness to participate in the trials with selected variables.

**Results:**

The study demonstrated 53% were less than 20 years old, 96% were single, and 22% lived in brothels. Eighty percent of the participants started selling sex at the age between 15 and 19 years old, 61% used illicit drugs for the first time when they were less than 20 years old, 24% had anal sex ever. Eighty-nine percent had more than 20-lifetime sexual partners, and 56% had unprotected sexual intercourse with sex clients. Ninety-one percent expressed a willingness to participate in the HIV vaccine trial. Sixty-one percent did not need permission from anyone for participating in a trial. Ninety-one percent expressed willingness to participate in the efficacy of HIV vaccine trial. In the logistic regression model, willingness was significantly associated with the need to ask permission for participation in HIV vaccine trial from sex agent.

**Conclusion:**

Respondent-driven sampling provided a rapid means of reaching young female sex workers who reported high-risk behaviors. The majority expressed a high level of willingness to participate in the HIV vaccine trial which was marginally correlated to the need to seek consent for participation in the trial from the sex brokers. Future HIV vaccine trials involving this population should consider involving the brokers in the trials because they form an essential part of the community for the participants.

## Background

Three decades have passed after the onset of HIV in most of the world including Tanzania. Currently, an estimated 34 million people living with HIV worldwide with sub-Saharan Africa remaining most severely affected [1]. Global efforts to strengthen HIV prevention and treatment programs have played a significant role in reducing the transmission of new HIV infection and AIDS-related deaths annually by 16 and 32% respectively by 2016. The steepest declines in numbers of new HIV infections between 2010 and 2016 were achieved in eastern and southern Africa (29% decline), Asia and the Pacific (13% decline), western and central Africa (9% decline), western and central Europe and North America (9% decline), the Caribbean (5% decline), and the Middle East and North Africa (4% decline) [1]. Since 2010, the new infections among young women and men globally (aged 15–24 years) have declined by 17 and 16% respectively by 2016. However, in high-prevalence settings like eastern and southern Africa, young women aged 15–24 years remain at unacceptably high risk of HIV infection, accounting for 26% of new HIV infections in 2016 despite making up just 10% of the population [1]. In Tanzania, the overall HIV prevalence has been declining from an estimated 5.7% in 2012 [2] to 4.7% among adults ages 15 to 64 years in 2016/2017 [3]. Prevalence among 15 to 24 years old is 1.4% with 2.1% among females and 0.6% among males by 2016/2017 [3]. This HIV prevalence among adults aged 15 years and older vary geographically across Tanzania, ranging from 11.4% in Njombe to less than 1 % in Lindi and Zanzibar [3].

A vast majority of HIV infections occur among populations at high risk for HIV transmissions such as people who inject drugs, sex workers, transgender people, prisoners, men who have sex with men and their sexual partners. Female, male and transgender sex workers, exist in all countries and often face significant vulnerability to violence, unsafe abortions, HIV and other STIs as a result of high-risk behavior, poor coverage by HIV and other health services, social marginalization, arrest and detention in closed settings, continuing stigma, discrimination, punitive laws, policies and practices [4]. Key populations and their sexual partners accounted for 80 and 25% of new HIV infections outside and in sub-Saharan Africa by 2015 underlining the importance of reaching them with services [1]. In Tanzania, HIV and STI Biological and Behavioral survey 2013 indicated that HIV prevalence among female sex workers was 26.6% [5]. A study in Zanzibar indicated the HIV prevalence among 15 to 29 years old female sex workers were found to increase from 6.4% in 2007 to 18.6% in 2011 [6]. Biomedical HIV prevention methods, as well as behavioral prevention, will be most effective if one considers structural and social factors, that increase risk and vulnerability, including gender inequality, HIV stigma and discrimination, and the social marginalization of the populations most at risk of HIV exposure [7]. In Tanzania, some HIV prevention interventions tailored to reach female sex workers had been in place. Such interventions included raising awareness of HIV prevention through implementation science research among FSWs with an introduction to oral pre-exposure prophylaxis to vulnerable young girls [8]. Providing linkages to health centers for contraceptives, HIV care, and treatment centers for pregnant and non-pregnant women and other STI services, condom use and HIV counseling and testing at the community level [9]. In the efforts to complement existing HIV preventive tools, Tanzania has participated in various HIV preventive biomedical trials. For instance, from 2009 to 2014 Tanzania has completed three early phase 2a HIV vaccine trials namely HIVIS 03, TAMOVAC 1 and TAMOVAC 2 among healthy HIV negative volunteers from the Police force and the general population. The outcome of those trials demonstrated that the vaccine candidates were safe and immune-potent [10]. The efficacious vaccine trials are now needed to test if the HIV vaccine could protect people from HIV infection. Ever since the outbreak of the HIV pandemic, of the six efficacy trials, conducted to date [11] only one, the RV144 Thai trial of ALVAC/gp120, showed protective efficacy [12]. In the quest to complement the current global and local HIV prevention efforts, our study aimed at understanding the recruitment approaches, risk behaviors, and practices and willingness of young female sex workers to participate in HIV efficacy vaccine trials. Furthermore, the study will shed a light on the complexities and dynamics of reaching vulnerable young women while highlighting factors affecting their willingness to participate in HIV vaccine clinical trials in Tanzanian settings.

### Ethical approval and consent to participate and for publication

The study received ethical approval by the Institutional Review Board at the Muhimbili University of Health and Allied Sciences with Reference number: 2017-04-12/AEC/Vol.XII/78. The study procedures were explained orally to each informant, followed by the signing of a consent form. The consent described the purpose of the study, voluntarism in participation with the right to refuse answering any question or withdrawing from the study at any time, the importance of participation and the benefits and risks of participating. The research assistants answered additional questions before each informant decided to sign the consent form. All participants provided their written consent at the start of the interviews. This manuscript does not contain any individual person’s data. At the end of the interview, each informant was reimbursed a total of 40,000 Tanzanian Shillings (equivalent to 18 United States of America dollars) as transport allowance to and from the study site as well as for compensation for 3 successful recruitment of their peers to the study.

## Methods

Study design: We conducted a cross-sectional study of 600 young female sex workers recruited by the respondent-driven sampling, RDS strategy. The study aimed to examine recruitment approaches, risk behaviors and willingness of young female sex workers to participate in an HIV vaccine trial.

Study site and population: The study took place in Dar es Salaam region at the Infectious Disease Clinic, IDC. The region has an estimated 4.4 million people [13]. It is one of the major cities with business, touristic and administrative importance in Tanzania. In this region, the HIV prevalence is at 4.7% which is slightly lower than the national prevalence estimated at 5% among adults ages 15 to 64 years [3]. In this region, various HIV preventive services are in place such as HIV counseling and testing, care and treatment, provision of contraceptive services and sexual reproductive health education to young people. IDC is a government facility located in the Ilala Municipality that provides youth-friendly health care services such as screening and treatment of sexually transmitted infections, contraceptives, counseling, testing, care and treatment for HIV, as well as general information about adolescent sexual and reproductive health [14]. The targeted population was young female sex workers aged between 18 and 25 years; who high-risk behaviors such as the history of any STI in the past 1 year, who had transacted sex in exchange for money, drugs, goods or gifts in the past 6 months before participation in this study. The young women who lived in Dar es Salaam for at least the past 6 months before the study and presented at the clinic with valid coupons. We excluded the participants who were either less than 18 or more than 25 years old and those without or with invalid coupons.

Sample size, sampling technique, and procedure: In this study, the participants were recruited from February to mid-May 2017 using respondent-driven sampling, RDS, a form of peer-driven chain referral sampling. This method reduced biases in recruitment by producing samples that were independent of the initial subjects from which sampling began [15]. Furthermore, it facilitated the recruitment of hard to reach participants by using a dual incentives system that compensated a participant for being interviewed and recruiting others in the study [15]. The findings of a formative study conducted among 15 informants between November and December 2016 using in-depth interviews set the foundation for the current study. The interviews gathered information on hotspots, scheduling of visits at trial centers, perceived stigma, and barriers to health-seeking behaviors. Initially, the researchers identified seeds or initial recruiters from 10 of 15 key informants. Eventually, the number of seeds increased to reach 20 because some initial recruitment chains died early by not referring their peers to the study site. The seeds recruited the first wave of study participants. Each of the seed and subsequent recruiters received three coupons with unique serial numbers. The researchers gave eligibility criteria to all recruiters as follows to recruit eligible female peers/friends, who were also exchanging sex for goods, gifts or money, whom they knew where they resided, to observe deadline for the validity of coupons given to recruited participants, of compensation as a recruiter after bringing eligible participant for enrolment in the research.

The sample size, *n* = 572 for the current study was calculated using the following formula *n* = [DE*Np (1-p)]/ [(d2 / Z2 1– α/2*(N-1) + p*(1-p)] by an OpenEpi, Version 3, open-source calculator. Definition of the formula is as follows: *N* = 6000, is estimated size of female sex workers population and p, 26.6% is the proportion of female sex workers infected with HIV in Dar es Salaam, d is standard of error, corresponding to 5%, and DE is design effect =2. For studies using respondent-driven sampling, the design effect of 2 is recommended to make a sample size twice as large as would be needed under simple random sampling. For this study, in order to account for seeds, missing data (drop-outs), non-response, and other sources of bias, the initial sample size above had been increased from 572 to 600 participants. In order to attain the required sample size, the research team distributed 1800 coupons, out of which a total of 650 participants were screened for eligibility. The study recruited 600 eligible participants out of 650 people screened for eligibility, including 20 participants who served as seeds during initial recruitment. Fifty participants, 8% were not included in the study because some came with expired coupons, were off age limit and others came after we stopped recruiting because we had reached the needed sample size.

At the clinic, each referred respondent voluntarily presented herself with a valid coupon, signed a consent form and filled the questionnaire. They have reimbursed a total of 18 United States of America dollars for being both study participants and recruiters. In order to minimize repeat enrolment among the study participants, the researchers recorded identifying information. That information, however, was not linked to questionnaires.

Data collection: Data collection was conducted using a self-administered questionnaire among 600 young female sex workers aged 18–25 years. In the current study, the questionnaires were prepared in English, translated to Kiswahili, and then back-translated to English for consistency. After signing of consent forms, the research participants filled in a questionnaire for assessing social network size, tracking of recruiter-recruit relationship and reasons for accepting the coupons. That process was followed by filling in of standardized behavioral assessment questionnaire for assessing risk behaviors, contraceptive uses, willing to participate in vaccine trials, utilization of health services, power dynamics within sexual networks concerning participation in vaccine trials and practices of using HIV preventive methods. For those who could not read and write, were asked to bring someone they trusted at the study site for assistance. After the participants submitted the questionnaires, research assistants reviewed the questionnaires to ensure the participants filled all questions correctly. Furthermore, the assistants provided clarified all questions that were not clear to the participants.

In our study tracking of recruiter-recruit relationship/recruitment patterns was done by documenting a unique identifying number of returned coupons, linking them with recruiter’s coupon unique identifying number. Like in RDS study conducted in United States of America, USA, the link between the recruiter and the recruit was documented by matching the serial numbers of the recruitment coupons given to each respondent with the serial numbers of the coupons returned to the project by the recruits [16]. All participants were counseled and tested for HIV only. The HIV testing followed The National HIV Testing algorithm that follows a ‘serial’ testing strategy. That is, a blood sample is first tested using a highly sensitive test, followed by a second highly specific test. A second test is only done when the first HIV test revealed an HIV- reactive result. HIV rapid testing can be performed in the laboratory or outside the laboratory setting by health service providers trained to performed HIV rapid tests [16]. The counselors referred to HIV positive participants to care and treatment centers of their own choice. The clinician provided a syndromic screening of other STIs to those requested. The counselors offered the participants condom, contraceptive pills and referral to the nearby health center as part of providing routine services.

Data management and analysis: In RDS, the sample is used to make estimates about social network connecting the population, then using information about social network size estimates about characteristics of the population are generated [17]. Estimates of weighted population proportions and 95% confidence intervals for select variables were calculated using the RDS-SS Analytical package. RDS-SS is a successive-sampling based estimator for population means based on respondent-driven sampling data when the estimated size of the target population is known. It addresses challenges of sampling without replacement in RDS researches [18]. Under successive sampling, larger units tend to be sampled earlier. This approach leverages the information in the decreasing size of sampled units overtime to make inferences about population size [19].

In the current study, we double entered the data to minimize entry error. We cleaned the data by comparing the unique RDS and registration numbers and other variables between the two data sets, data discrepancies identified were resolved by going back to the source documents (questionnaires). After that, coding of some variables into binary and categorical variables was done, with analytical outputs of weighted estimates of the population proportions of selected variables of interest. Our proposed estimator is based on estimated sampling weights [10], which are based on the sampling probability given by the successive sampling procedure, applied to nodal sampling units and sizes given by nodal degrees [19]. Furthermore, STATA analyzed weighted data, taking account of the weighting and clustering. The associations of willingness to participate in HIV vaccine trials using selected independent variables were determined through computing weighted data by STATA 13/SE package in regression models [20]. The variables with a *P*-value of ≤0 .2 were entered into the final model to look for factors associated with willingness to participate in trials, WTP. The odds ratios with 95% confidence intervals used to present the results of logistic regression models. After that, all variables that had a *P* value of ≤0.05 were regarded as statistically significant in the final model.

## Results

### Recruitment

**Fig. 1:**
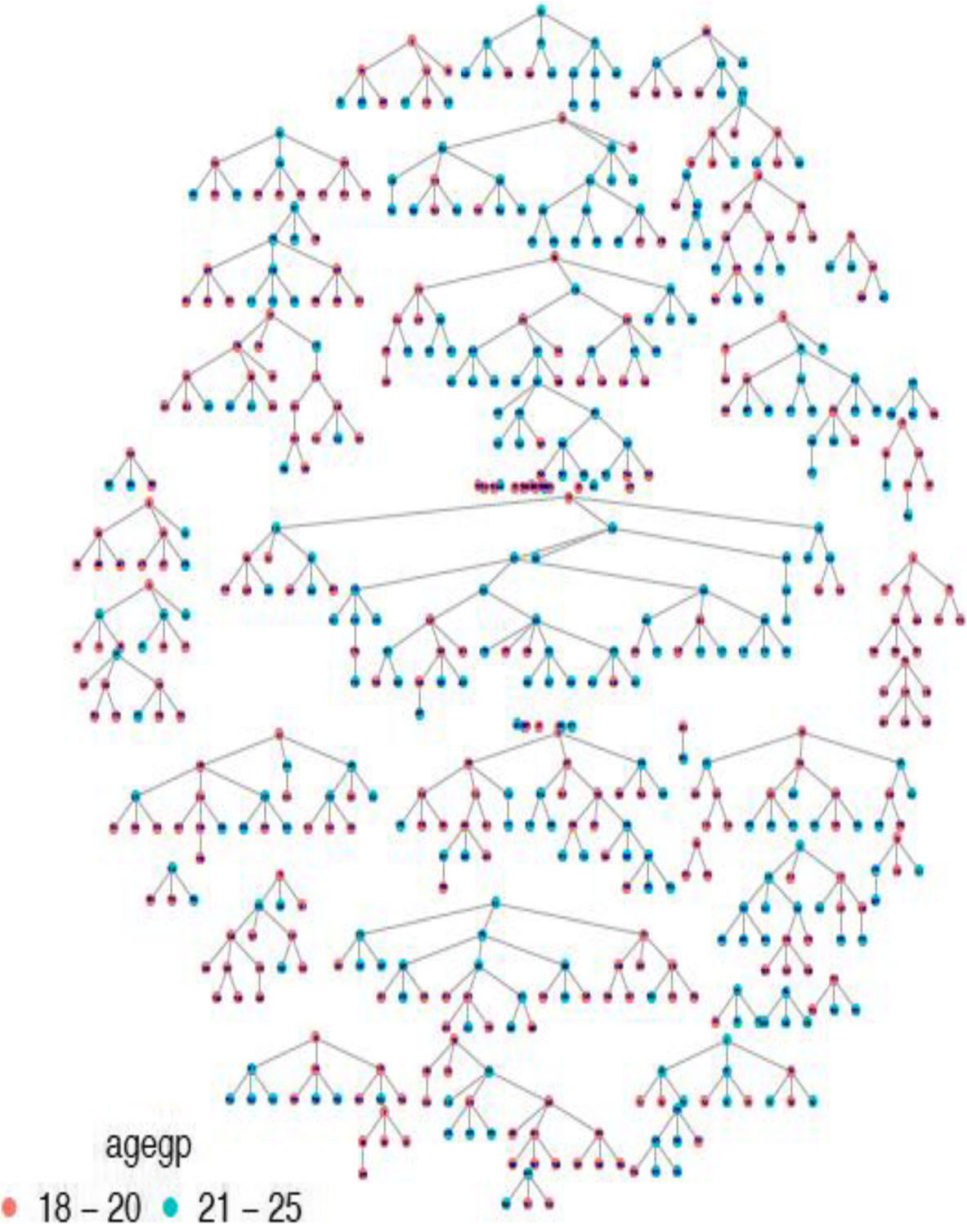
It shows recruitment pattern by age group and network size of young female sex workers, FSWs

The enrollment of the participants took place between February and May 2017. At the beginning of the study, ten seeds were recruited, yielded fewer than 50 participants over the first month. The attrition rate was 79% in the 1st months of recruitment by 10 seeds. As a result, a total of 20 seeds were recruited for faster recruitment to occur. Some of the reasons for the failure of early seeds to recruit were speculated to be fear of being caught by authority figures, inadequate information on recruitment given to the participants, and not being interested in the participation of study. For this study, we used peers to recruit other peers, that assured participants of their safety and anonymity. There was a maximum of 7 waves of recruitment, most seeds recruiting 3 participants in the study. The participants were able to recruit their peers regardless of age, i.e. young FSWs recruited older FSWs and vice versa. Most participants reported network size ranging from 1 to 10, Fig. 1 below.

### Sociodemographic characteristics

Table 1 depicts the sociodemographic characteristics of the participants using unweighted and weighted frequency distribution of variables. The respondents had an age range of 18 to 25 years with a median age of 20 years. Among 600 respondents, slightly more than half, 53% reported being less than 20 years old. A total of 240 (39%) reported being residing in Temeke municipality, with most of the respondents, 48% reporting to be living in the same residence for more than 12 months. The majority of the participants 577 (96%), reported to be single, 264 (44%) and 133 (22%) lived with peers and in brothels respectively. More than 60% of 600 respondents had finished primary school education.

**Table 1:** Sociodemographic characteristics of young female sex workers in Dar es Salaam

Variables	Weighted frequency %,	n	N
	(95% CI)		
Age (years)			600
18–20	53.1(0.48–0.58)	313	
21–25	46.9(0.42–0.52)	237	
District of Residence			600
Temeke	39(0.33–0.45)	240	
Ilala	21(0.17–0.25)	123	
Kinondoni	20(0.17–0.25)	115	
Kigamboni	19(0.13–0.24)	113	
Ubungo	1(0.00–0.03)	9	
Marital status			598
Single	96.30(0.94–0.99)	577	
Divorced/widow	2.3(0.01–0.04)	14	
Married/cohabiting	1.4(−0.003–0.03)	7	
Level of education			600
No formal education	17.2(0.139–0.204)	107	
Primary education	60.7(0.57–0.65)	364	
Ordinary Secondary	21.1(0.18–0.25)	124	
Vocational schools	0.7(−0.0004–0.01)	3	
University	0.4(−0.002–0.01)	2	
People participants were living with			600
Peers	43.69(0.39–0.48)	264	
Brothel	21.6(0.18–0.25)	133	
Parents/guardians	19.9(0.15–0.24)	112	
Alone	12.8(0.1–0.16)	79	
Spouse/partners	1.2(0.003–0.02)	7	
Sex agent/Pimp	0.9(0.001–0.02)	5	
Period of Living in current residence			598
0-6 M	16.6(0.14–0.20)	95	
6-12 M	35.4(0.32–0.39)	203	
> 12 M	48(0.44–0.52)	300	

### Risky behavioral practices

Table 2 shows 26% of the respondents had their first sexual encounter at the age of 14 years and below. Most of the respondents, 80% stated to have started selling sex at the age between 15 and 19 years old. When interviewed about reasons for selling sex, 54% mentioned it was a primary source of income, 20% mentioned peers’ pressure, and 13% stated they were orphans without other means of support. A vast majority of respondents, 519 (87%), stated they had no other means of earning income apart from sex work. More than two thirds, 63%, reported meeting clients in recreational places like bars and hotels. Eighty-nine percent had more than 20-lifetime sexual partners, with 30% reported to have more than five clients on the last day of business. Almost a quarter, 24%, of the participants, reported to ever had anal sex. Also, slightly more than half, 56%, of the participants had unprotected sexual intercourse with sex clients. Of 343 respondents who reported having unprotected sexual intercourse with the clients, 52% cited the reason as clients’ refusal to use the condom, 13% mentioned not thinking of using the condom, and 10% mentioned not liking using condoms. Two thirds, 61%, of the participants used illicit drugs for the first time before 20 years of age, with 86% reported to have used illicit drugs for more than 24 months prior to the study. The majority reported marijuana as the most commonly used recreational illicit drug. A vast majority, 77%, reported having had taken alcohol in the lifetime, with 73% of respondents admitting to having drunk alcohol before sex with clients. When asked about power dynamics in their sex business, only 185 (31%), of the respondents admitted to having had an agent for selling sex; 118 (44%), reported having sex agent controlling or influencing their income from sex work.

**Table 2:** Frequency distribution of sexual behaviors among young female sex workers in Dar es Salaam

Variables	Weighted frequency %	n	N
	(95 CI %)		
Alcohol Intake			600
Yes	76.7(0.73–0.80)	464	
No	23.3(0.20–0.27)	136	
Alcohol intake during sex			490
Yes	72.7(0.68–0.77)	358	
No	27.3(0.23–0.32)	132	
Have ever used illicit drugs			
Yes	20.5(0.17–0.23)	122	596
No	79.5(0.76–0.83)	474	
Have had anal sex			
Yes	23.7(0.20–0.27)	142	599
No	76.3(0.73–0.80)	457	
Have had unprotected sex with an irregular client			
Yes	55.8(0.52–0.60)	334	599
No	44.2(0.40–0-48)	265	
Having an agent for soliciting sex clients			
Yes	31(0.27–0.35)	185	597
No	69(0.65–0.73)	412	
Having income apart from selling sex			599
Yes	13.4(0.11–0.16)	80	
No	86.6(0.84–0.89)	519	
Age when started to use illicit drugs for the first time			118
10–14 years	14(0.08–0.21)	15	
15–19 years	61(0.51–0.72)	74	
20–25 years	25(0.16–0.33)	29	
Age at 1st sexual intercourse			
≤ 14 years	26.4(0.23–0.30)	157	593
> 14 years	73.6(0.70–0.77)	436	
Reported number of lifetime sex partners			
≤ 20	11(0.09–0.14)	65	600
> 20	89(0.86–0.91)	535	
Number of sexual clients at last day of business			
≤ 5	70(0.66–0.73)	407	600
> 5	30(0.27–0.34)	193	
Period of using illicit drugs in life time			
≤ 24 months	14.5(0.11–0.17)	89	600
> 24 months	85.5(0.83–0.89)	511	
Age when started selling sex			
10–14 years	9(0.83–0.89)	54	600
15–19 years	80(0.77–0.83)	480	
More than 20 years	11(0.09–0.14)	66	

### Willingness to participate in an HIV vaccine trial

Table 3 depicts that almost all respondents, 545 (91%), responded positively to a question on willingness to participate in a hypothetical HIV vaccine trial. The main reason given for wanting to participate in an HIV vaccine trial was altruism, wanting to contribute to a cure for HIV and wanting to learn about vaccine trials. Very few, 1%, had taken part in a scientific trial. The majority were able to recite different HIV prevention methods such as 61% reported about behavioral changes interventions, 15% stated preventive HIV clinical trials and 12% mentioned the use of pre-exposure prophylaxis. When asked about things that would make young females come for follow up during the trial, 36% of respondents mentioned the presence of investigational services, 20% reported altruism and, 16% mentioned the presence of health insurance services.

**Table 3:** Bivariate and multivariable logistic regression for factors associated with willingness to participate in HIV vaccine trial, WTP

Factors	WTP	Crude OR (95% CI)	*P* value	Adjusted OR (95% CI)	*P* value
	n (column%)				
Need to ask permission from sex agent for participation in HIV vaccine trial					
Yes	131(24%)	0.60(0.32–1.15)	0.13	0.46(0.23–0.94)	0.04
No	412(76%)	1		1	
Participation in HIV vaccine trials may affect relationship with clients					
Yes	226(41%)	1.55(0.83–2.90)	0.17	1.50(0.79–2.85)	0.2
No	319(59%)	1		1	
Influence of client during participation in HIV vaccine trial					
Yes	90(17%)	0.57(0.29–1.13)	0.11	0.70(0.36–1.32)	0.3
No	455(83%)	1		1	
Having income apart from selling sex					
Yes	69(13%)	0.58(0.27–1.26)	0.17	0.59(0.27–1.30)	0.2
No	475(87%)	1		1	

^1^Bivariate analysis between all independent variables and willingness to participate in efficacy HIV vaccine trial was done. Only few variables with significance level at *p* ≤ 0.25 were included in multivariable analysis

Sixty-one percent, 61%, of the participants did not need permission from anyone for participating in a trial. Those who reported being needing permission for participation mentioned parents and guardians as crucial people for seeking permission. Eighty-one percent of 600 reported not to need permission for participation in HIV vaccine trials from sex clients. Nearly two-thirds of 600 respondents, 59%, did not think being in the HIV vaccine trial would affect their relationship with clients. A vast majority, 83, and 86% did not think clients and sex agents would not have any improper influence on participation in the HIV vaccine trials respectively. In a bivariate analysis of factors contributing to a willingness to participate in HIV vaccine trials with explanatory variables such as sociodemographic characteristics, risk behaviour, having someone to influence the decision for participation, only three variables with *p*-value ≤0.25 were included in the multivariable logistic regression model. In the final regression model, the likelihood of the willingness to participate in the efficacy of HIV vaccine trials was not influenced by any independent variable.

## Discussion

This study offers an understanding of means of recruitment, characteristics of the participants, risk behavior practices and other social dynamics existing in a population of vulnerable young female sex workers in DSM concerning participation in HIV vaccine trials in Tanzanian settings. This study demonstrated that the female sex workers at high risk for HIV could be reached and possibly recruited for HIV prevention studies including preventive HIV vaccine trials. Besides, the participants had shown a high level of willingness to participate in the hypothetical HIV vaccine trial. Recruitment using the approach of RDS saved time, by successfully recruiting the participants within 4 months despite the presence of stigma and fear attached to being female sex workers in Tanzanian settings. Compared to recruitment in TaMoVac studies, in which multistage recruitment approach was applied [21], in the current study, only single stage recruitment approach was used, that enabled researchers to reach young female sex workers for the current study.

Furthermore, our findings demonstrated the young female sex workers were able to recruit other regardless of the age of recruiter, level of education, and participants’ network sizes, showing RDS could reach participants with diverse characteristics within a reasonable recruitment period. Our findings were similar to a study done in New York City among drug users; their results suggest that RDS provides an effective method for recruiting a large sample of drug users for conducting HIV-related behavioral surveys in a large city [22]. That study demonstrated the substantial level of cross-gender and inter-ethnic mixing facilitated the emergence of diversity within each recruitment chain by reducing the number of waves required for equilibrium to be approximated [22]. Furthermore, a vast majority of females in the present study indicated that they had stayed in current residence for more than 12 months indicating some level of communal connection and stable settlement. This information could play a crucial role in decision making among researchers about whether or not to engage FSWs, particularly in lengthy HIV vaccine efficacy trials. In such trials, retention should be optimum for close monitoring of the progress of trial participants and getting desirable trial outcomes. In many biomedical HIV prevention studies, the researchers regarded the young FSWs as a highly mobile population despite demonstrating satisfactory rates of retention in studies. An observational cohort of female sex workers, FSWs in South Africa demonstrated 86% retention rates for the HIV-negative FSWs throughout follow-up despite the highly mobile nature of that population. In that study, the researchers thought a dedicated network of community liaisons and efforts to engage the community on a structural level highly contributed to their retention in the study [23].

### Risk behavior practices

The HIV prevalence was very low, 2.2% in a sample of 600 participants at high risk for HIV infection. This prevalence is approximately the same as that of young females in the general population aged 15 to 24, estimated at 2.1 [3]. Similarly, in a study done to assess HIV risk and willingness to participate in HIV vaccine trials in three high-risk populations; men having sex with men, intravenous drug users and female sex workers in Spain observed a low HIV prevalence of 3.8% among 844 individuals [24]. The low HIV prevalence observed in our study population may be attributed to decreased in HIV prevalence in Dar es Salaam [3] existing biomedical and behavioral HIV preventive interventions among sex workers such as condom use, post-exposure prophylaxis, widely use of antiretroviral drugs that play vital role in viral load suppression and presence of community outreach programs that have been explicitly designed to reach FSWs. In those programs, the researchers visited FSWs during the night hours at hot spots such as bars, roads, and hotels to offer services such as HIV testing and counseling, linkage services for HIV infected individuals, STI screening and treatment [9].

Despite the low prevalence of HIV observed, most of our study participants demonstrated risk behavior practices that may continually predispose them to HIV infection because they considered sex business as their primary means of earning income with no clear evidence of having an alternative means of earning a living. Such practices included selling sex and use of illicit drugs at a young age, having unprotected sexual intercourse with sex clients, many lifetime sex partners and practicing anal sex. Similar characteristics were observed among black South African women who used transactional sex as a mode of economic survival, hence putting them at higher risk for HIV and another infectious disease [25].

However, this study demonstrates that in situations where other functioning HIV preventive activities are in place, having high-risk behaviors alone may not be enough or a reliable indicator for the suitability of the participants to participate in efficacy HIV vaccine trials. It is therefore imperative to carry out an accurate HIV incidence study to ascertain the suitability of high-risk young females in the participation of the 3rd phase HIV vaccination trials.

### Willingness to participate in HIV efficacy vaccine trials

This study demonstrated the majority of the participants showed the willingness to participate in HIV vaccine trial at phase III mainly because of altruism. This willingness to participate may suggest that female sex workers were open to participating in various activities to prevent HIV transmission. Our participants valued their contribution and felt they were an integral part of the solution to a bigger problem of the HIV burden in our settings. Several studies assessing the preparedness of high-risk groups in HIV vaccine trials have documented a high level of WTP. In Spain, 82% of the three high-risk populations demonstrated expressed their willingness to participate in HIV vaccine trials in the study [24]. Likewise, the vast majority, 88.9%, of enrolled women reported that they would be willing or probably willing to participate in a future HIV vaccine trial. In that study, significant factors associated with willingness to participate were perceived personal benefit from an HIV vaccine, a client forced the woman to have sex, and the woman felt she could become infected with HIV in the following 5 years [26].

Strikingly, the need to have consent from sex agents/brokers were moderately correlated with the likelihood of the participants to participate in the HIV vaccine trials. This may imply the brokers form an integral part of the community surrounding the participants, as a result, the brokers may be influential entities when the time comes for the study participants to decide whether or not to engage in HIV vaccine trials. This calls for further investigations to iron out types of influences posed by the brokers and how they might affect the whole process of participation of the trials among young female sex workers. This dependency on some kind of members of the community for decision making among the current participants was supported by a study in Dar es Salaam [27]. In that study, members of the community thought that preventive biomedical interventions required support from and acceptance by the community since potential young participants in HIV interventions depend on members of their community for social and sometimes even economic support [27]. In contrary, most of the study participants showed a certain degree of independence from parents/guardians as far as the trial participation is concerned, suggesting that the participants in the current study were exposed to hard social and economic challenges from a very early age, pushing them to bond with people like the sex agents whom they interacted frequently in their social environment. This highlights the further critical role the brokers might be playing around decision-making process among young female sex workers.

Even though our study participants demonstrated a high level of risky sexual behaviors, those behaviors as well as age, level of education and marital status did not support the possibility of willingness to participate in the HIV vaccine trial. Our findings may cautiously suggest that our study participants might have less complicated influencers that would negatively influence their participation in the HIV vaccine trials.

## Conclusion

RDS provided rapid means of reaching young female sex workers with high-risk behaviors that might predispose to HIV infection. They expressed a high level of willingness to participate in the HIV efficacy vaccine trial which was moderately influenced by the need to seek consent from the sex brokers. It is important to study further types of influence on decision making exerted by the brokers for successful recruitment and participation of young female sex workers in the HIV vaccine trials.

## Methodological considerations

This study recruited 600 young female sex workers using RDS. The HIV prevalence examined in this group was lower than what has that prevalence of the FSWs documented elsewhere in Tanzania. Therefore, understanding and interpretation of reported risk high behaviors among the study participants should be done with caution, and may not be generalized in other settings. However, the presence of stable and functional HIV preventive programs at the community level in Dar es Salaam, as well as a lowered HIV prevalence reported recently in the same region may explain the low HIV prevalence among those who participated in the study. Secondly, it is also possible those who chose to participate in the study at IDC knew their negative HIV status, and the ones who knew their positive HIV status refused to participate because of real stigma in the society. However, since the primary aim of the study was not to elicit HIV status among the study participants, instead of assessing their risk behaviors, our findings can be used to inform other researchers within a similar context as our settings. HIV incidence studies should be carried out in order to establish a true HIV burden among the key populations to determine their actual suitability for participating in clinical HIV efficacious study.

## Data Availability

All data generated and analyzed during this study are included in this article.

## Abbreviations

AIDS: Acquired Immunodeficiency Syndrome
FSW: Female sex worker
HIV: Human Immunodeficiency Virus
HIVIS 03: HIV Immunogenicity Study 03
IDC: Infectious Diseases Clinic
RDS: Respondent driven sampling
RDS-SS: Respondent driven sampling of successive sampling
RV 144: Acronym for Thailand HIV vaccine trial
STI: Sexually transmitted infection
TAMOVAC: Tanzania Mozambique Vaccine trial project
WTP: Willingness to participate
